# Protocol for the synthesis and characterization of Ru@PCN@PEI nanoparticles with superoxide dismutase catalytic activity

**DOI:** 10.1016/j.xpro.2026.104412

**Published:** 2026-03-05

**Authors:** Meijing Wang, Tao Chen, Xinyi Zeng, Jiasen Lin, Yan He, Xujie Liu

**Affiliations:** 1 School of Biomedical and Pharmaceutical Sciences, Guangdong University of Technology, Guangzhou 510006, China

**Keywords:** Biotechnology and bioengineering, Chemistry, Material sciences

## Abstract

Bimetallic organic frameworks (BMOFs) possess versatile structural and functional features that enable applications in catalysis and biomedicine. Here, we present a protocol to synthesize Ru@PCN@PEI nanoparticles by loading Ru nanoparticles onto PCN-222(Mn) and coating the surface with polyethyleneimine (PEI). We describe steps for precursor preparation, particle size regulation, and surface modification. We detail procedures for morphological characterization by transmission electron microscopy and assessment of catalytic activity using ultraviolet-visible (UV-vis) spectroscopy. This protocol enables the preparation and functionalization of BMOFs for advanced nanobiomaterial applications.

## Highlights

• Optimize PCN-222(Mn) NP size by adjusting the ZrOCl2:BA ratio
• Steps for synthesizing Ru@PCN@PEI NPs by loading Ru and coating with PEI
• Guidance on the characterization of Ru@PCN@PEI NPs using TEM, SEM, and DLS
• Procedures for evaluating SOD-like activity of Ru@PCN@PEI NPs via UV-vis

## Abstract (editor highlights)

Publisher’s note: Undertaking any experimental protocol requires adherence to local institutional guidelines for laboratory safety and ethics.

## Graphical abstract

**Figure:**
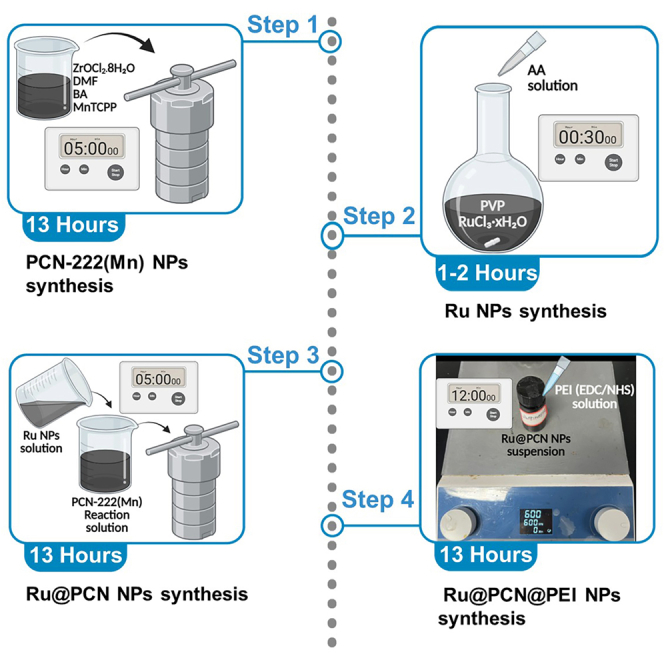

## Before you begin

BMOFs combine the structural advantages of metal-organic frameworks with the catalytic activity of metal catalysts, offering great potential in mimicking the catalytic functions of natural enzymes.^1^ Studies have shown that PCN-222(Mn) nanoparticles (NPs) combines the stability of zirconium (Zr) and the high catalytic activity of manganese (Mn), exhibiting superoxide dismutase (SOD)-like activity.^2^ However, the current PCN-222(Mn) NPs prepared have relatively large particle sizes (>200 nm), which limits their application in the biomedical field.^3^^,^^4^ Therefore, controlling the particle size of PCN-222(Mn) and modifying it to enhance its SOD-like activity is of significant importance.^5^

This protocol describes the synthesis of Ru@PCN@PEI NPs with tunable particle size and superoxide dismutase (SOD)-like catalytic activity. The workflow includes the solvothermal synthesis of PCN-222(Mn) NPs with the aid of modulators to achieve the desired particle size, followed by in situ incorporation of Ru NPs to obtain Ru@PCN NPs, and a surface coating with polyethyleneimine (PEI). Although this work focuses on Ru NPs and PEI, the same principles can be extended to other metal species, framework compositions, and polymer coatings to guide the design of functional BMOF-based materials.

### Innovation

This protocol provides a systematically optimized procedure for the fabrication of Ru@PCN@PEI nanoparticles, starting with the synthesis of size-tunable PCN-222(Mn) as a foundational framework. Previous reports often produced large microcrystals or provided limited guidance on modulator dosage. To address this, we establish a refined operational range for benzoic acid (BA), providing practical reference conditions for precursor optimization to consistently achieve uniform products within a sub-200 nm size regime.

While in-situ incorporation of pre-formed nanoparticles during MOF synthesis is well-established, this protocol represents the first application of this approach to Ru NP incorporation in PCN-222(Mn) frameworks. This design avoids harsh in-framework reduction, minimizes structural damage, and can be extended to load other preformed metallic NPs into porous BMOF systems.

Moreover, the protocol introduces a mild aqueous PEI modification using EDC/NHS activation, enabling uniform surface coating without thermal treatment. Combined with systematic purification and staged freeze-drying, the workflow offers a reproducible path from precursor synthesis to catalytic evaluation, enhancing reproducibility, scalability, and adaptability for BMOF-based nanomaterials.

### Clean glassware

**Timing: 1 h**

1. Freshly clean all glassware and PTFE liners with aqua regia (HNO_3_: HCl = 1:3, v/v), rinse thoroughly with deionized water, and dry at 60–80°C or allow to air dry.

**CRITICAL:** Handle aqua regia only in a certified fume hood with full PPE. Do not expose stainless-steel parts to aqua regia.

## Key resources table

**Table:** 

REAGENT or RESOURCE	SOURCE	IDENTIFIER
**Chemicals, peptides, and recombinant proteins**		

Zirconyl chloride octahydrate (ZrOCl_2_·8H_2_O, ≥98%)	BIDE	CAS: 13520-92-8
Benzoic acid (BA, ≥99%)	Macklin	CAS: 65-85-0
N,N-Dimethylformamide (DMF, 99.8%)	Aladdin	CAS: 68-12-2
MnTCPP (MnTBAP, ≥98%)	BIDE	CAS: 55266-18-7
Ruthenium(III) chloride hydrate (RuCl_3_·xH_2_O, 99.9%)	BIDE	CAS: 14898-67-0
Polyvinylpyrrolidone (PVP, Mw = 58,000)	Aladdin	CAS: 9003-39-8
Ascorbic acid (AA, ≥99%)	Aladdin	CAS: 50-81-7
Polyethyleneimine (PEI, branched, Mw = 1800)	Aladdin	CAS: 9002-98-6
1-Ethyl-3-(3-dimethylaminopropyl)carbodiimide hydrochloride (EDC·HCl)	Aladdin	CAS: 25952-53-8
N-Hydroxysuccinimide (NHS)	Aladdin	CAS: 6066-82-6
1,2,3-Benzenetriol (Pyrogallol, ≥99%)	BIDE	CAS: 87-66-1
2-Amino-2-(hydroxymethyl)propane-1,3-diol (Tris, ≥99%)	BIDE	CAS: 77-86-1
Ethylenediaminetetraacetic acid disodium salt (EDTA disodium salt, ≥99%)	Aladdin	CAS: 139-33-3
Hydrochloric acid (HCl, 37%)	Aladdin	CAS: 7647-01-0

**Other**		

Freeze dryer	Sihuan Furui	LCMS-2020
Magnetic stir plate	SUNNE	SN-MS-6D
High-speed refrigerated centrifuge	Xiangyi	JIDI-20D
Micropipettes, Research plus P200 and P1000	Eppendorf	Cat#3120000056 Cat#3120000080
Transmission electron microscope (TEM, 120 kV)	Hitachi	HT7700
UV–visible spectrophotometer	PerkinElmer	Lambda 25
Analytical balance	OHAUS	FA1004
Convection oven	Yiheng	N/A
Ultrasonic bath	Kedao	SK7200LHC

## Materials and equipment

**Table:** Stock solution of ascorbic acid (AA) reducing agent

Reagent	Final concentration	Amount
Ascorbic acid	100 mM	179.8 mg (1.02 mmol)
Deionized water (DI water)	N/A	Adjust to a final volume of 10 mL
Total	100 mM	10 mL

***Note:*** Store this solution in the fridge at 4°C for 1 month.

**Table:** Stock solution of polyethyleneimine (PEI) with EDC and NHS

Reagent	Final concentration	Amount
Polyethyleneimine (branched, Mw = 1800)	139 mM	500.85 mg (0.278 mmol)
1-Ethyl-3-(3-dimethylaminopropyl)carbodiimide hydrochloride (EDC·HCl)	50 mM	19.2 mg (0.100 mmol)
N-Hydroxysuccinimide (NHS)	50 mM	11.54 mg (0.100 mmol)
Deionized water (DI water)	N/A	Adjust to a final volume of 2 mL
Total	N/A	2 mL

***Note:*** Store this solution in the fridge at 4°C for one day.

## Step-by-step method details

### Synthesis of size-tunable PCN-222(Mn) NPs

**Timing: 13 h**

Synthesize PCN-222(Mn) nanoparticles with tunable sizes by adjusting the ratio of ZrOCl_2_·8H_2_O (Zirconyl chloride octahydrate) and benzoic acid (BA).

1. Weigh 60 mg of ZrOCl_2·_8H_2_O (0.186 mmol) and add it to a 50 mL glass beaker.

***Note:*** ZrOCl_2_·8H_2_O is hygroscopic. To ensure accurate weighing of the zirconium precursor, store the reagent in a dry environment and seal the bottle tightly with Parafilm immediately after each use.

2. Using a 50 mL graduated cylinder, measure 20 mL N,N-dimethylformamide (DMF) and add it to the beaker.
3. Sonicate the mixture for 5 min until the zirconyl salt is fully dissolved.
4. Select the appropriate amount of BA according to Table 1 to achieve the desired nanoparticle size.
  a. Weigh the selected amount of BA and add it to the mixture.
  b. Sonicate for 5 min until fully dissolved to obtain a homogeneous solution.

**Table 1:** Influence of benzoic acid (BA) concentration on the size of PCN-222(Mn) nanoparticles(n=3)

ZrOCl_2_: BA Ratio	BA (mg)	Z-average (nm, mean±SD)
1:25	580	310.83 ± 5.49
1:22	510.4	145.10 ± 3.75
1:20	464	138.20 ± 2.59
1:18	417.6	106.20 ± 2.96
1:16	371.2	82.38 ± 0.52
1:14	324.8	60.10 ± 1.75
1:12	278.4	34.80 ± 1.21
1:10	232	104.20 ± 1.49
5. Weigh 20 mg of MnTCPP and add to the solution. Sonicate for 5 min to obtain a deep-black, homogeneous solution.

**CRITICAL:** Maintain the consistency of reagent addition order to avoid premature reactions due to differing contact sequences.

***Note:*** During sonication, an ice pack can be added to the sonicator to prevent overheating.

6. Transfer the mixture to a 50 mL PTFE liner and place the liner in a stainless-steel autoclave.
  a. Tighten the safety cap with the supplied rod.
  b. Incubate the autoclave in a 90°C oven for 5 h to obtain a deep-green solution.
7. After the reaction, allow the autoclave to cool to room temperature (25°C ± 1°C). Transfer the solution to a 50 mL centrifuge tube for subsequent centrifugation-based purification.

***Note:*** Wear heat-resistant gloves to carefully transfer the autoclave into the oven to avoid burns and potential explosion. After the reaction, first turn off the oven, open the oven door to dissipate heat for 1 h. After 1 h, carefully remove the autoclave using heat-resistant gloves and place it in a safe area. Allow it to cool naturally for at least 6 h before opening the autoclave to prevent explosion due to high temperature and pressure.

### Characterization of PCN-222(Mn) NPs

**Timing: 1–2 h**

**Figure 1:**
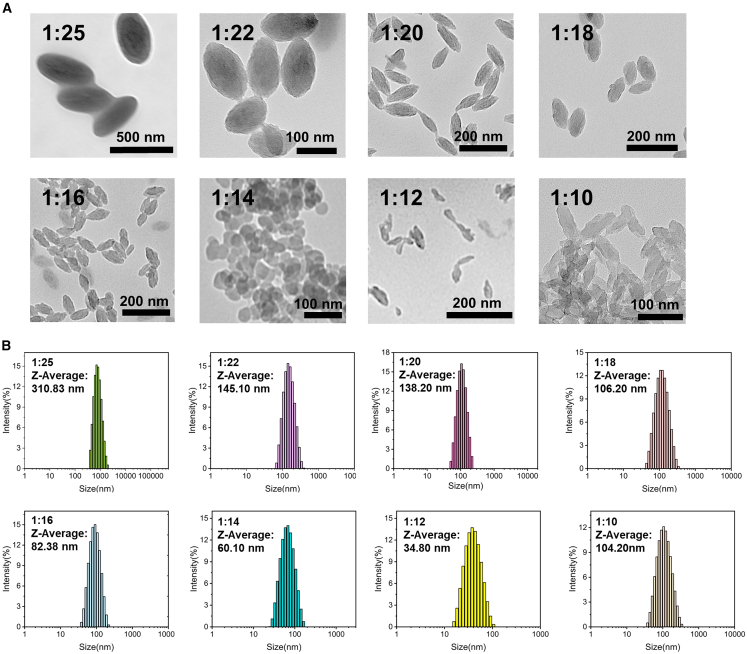
Characterization of PCN-222(Mn) NPs prepared with different ZrOCl_2_: BA ratios (A) TEM imaging of PCN-222 (Mn) NPs with different ZrOCl_2_: BA ratios. (B) DLS imaging of PCN-222 (Mn) NPs with different ZrOCl_2_: BA ratios (n=3).

After the successful preparation of size-tunable PCN-222(Mn) NPs, transmission electron microscopy (TEM) and dynamic light scattering (DLS) are used to characterize the particle size and morphology of PCN-222(Mn) NPs (Figure 1).

8. Disperse 1 mg PCN-222(Mn) NPs into 1 mL DI water and sonicate for 5 min to form homogeneous dispersion.
9. Drop 20 μL of the PCN-222(Mn) NPs dispersion on the polysilicon wafer, then transfer the polysilicon wafer to oven, drying for 1 h at 60°C for further TEM characterization.

***Note:*** TEM imaging should be performed by a trained technician or engineer following standard operating procedures.

10. Add 3 mL of DI water to the solution from step 8, mix by pipetting with a 1 mL pipette, then transfer the solution to a DLS cuvette for DLS measurement on the instrument.

### Synthesis of Ru NPs

**Timing: 1–2 h**

Synthesize Ru nanoparticles stabilized by PVP via aqueous reduction at elevated temperature.

11. Using an analytical balance, weigh 200 mg of PVP (Mw = 58,000) and 15 mg of RuCl_3_·xH_2_O into a 100 mL round-bottom flask.
12. Using a graduated cylinder, measure 60 mL deionized water and add it to the flask to prepare the precursor mixture.
13. Place the precursor mixture in an oil bath maintained at 115°C, and stir at 30 rpm. Perform the reaction away from direct sunlight.

***Note:*** Wear protective equipment and handle the 115°C oil bath with care. The stirring rate of 30 rpm provides gentle mixing to avoid excessive shear. If the solution is uneven, the stirring rate can be slightly increased.

14. Prepare a 0.10 M ascorbic acid (AA) solution: weigh 0.1798 g AA (1.02 mmol) and dissolve it in 10 mL of deionized water. Sonicate for 3 min until fully dissolved.
15. Using a pipette, slowly add 1 mL of the ascorbic acid solution dropwise to the precursor mixture over 1–2 min while maintaining the temperature at 115°C.
16. After the addition, continue the reaction for 30 min. The solution will gradually darken, yielding the Ru nanoparticle suspension (refer to Figure S1).

***Note:*** Both AA and Ru compounds are prone to oxidation; prepare solutions and conduct reactions under low light conditions whenever possible.

### Purification and freeze-drying of Ru NPs

**Timing: 6–7 days**

The following steps outline the purification and freeze-drying process for Ru nanoparticles, which ensures the removal of unreacted reagents and solvents, resulting in pure Ru NP powder suitable for further applications.

17. Transfer the Ru NPs suspension into pre-rinsed 20 kDa dialysis bags.
18. Place the bags in a beaker containing an excess volume of DI water, protecting the solution from light, and dialyze for 4 days.

***Note:*** The volume of liquid in the dialysis bag should not exceed half of its total capacity to avoid affecting dialysis efficiency.

19. Replace the external DI water 3–4 times per day to maintain the concentration gradient and ensure efficient dialysis.

***Note:*** Dialysis is a reliable method to ensure thorough purification. While this step typically requires 4 days, readers can shorten the process to 2–3 days by increasing the frequency of water changes from 3–4 times per day to 6–8 times per day to accelerate the removal of impurities.

20. Collect the dialyzed solution and transfer it to 50 mL centrifuge tubes.
21. Centrifuge at 15,292 × g (12,000 rpm), 4°C for 30 min. Discard the supernatant and retain the pellet.

***Note:*** Maintaining the centrifuge at 4°C is a standard precautionary measure to mitigate potential temperature increases during high-speed rotation (15,292 × g).

22. Freeze the pellet at −80°C for 12–16 h.
23. Place the frozen pellet in a lyophilizer and lyophilize (condenser temperature −60 °C and vacuum pressure < 15 Pa) for 2–3 days to obtain Ru NPs powder.
24. Aliquot the powder into microcentrifuge (EP) tubes, label, and store protected from light until use.

***Note:*** Sufficient pre-cooling (at least 12 h at −80°C) before freeze-drying can prevent the formation of large ice crystals, which helps maintain the dispersion of nanoparticles. Before using freeze-dried Ru NPs, sonicate in deionized water (avoid excessive sonication exceeding 10 min).

***Note:*** The final product should be a fine black powder. Upon re-dispersion in DI water followed by 3 min of sonication, it should form a deep-black suspension.

### Synthesis of Ru@PCN NPs

**Timing: 13 h**

Synthesize Ru@PCN NPs via a one-pot method by incorporating Ru NPs into the pores of PCN-222(Mn) during the synthesis of the PCN precursor solution.

25. Weigh 60 mg of ZrOCl_2_·8H_2_O (0.19 mmol) and add it to a 50 mL beaker.
26. Using a 50 mL graduated cylinder, measure 15 mL DMF and add it to the beaker to prepare the the precursor solution.
27. Sonicate the precursor solution for 5 min until ZrOCl_2_·8H_2_O is completely dissolved.
28. Weigh 464 mg of benzoic acid (BA, 3.80 mmol), add to the precursor solution, then sonicate for 5 min to obtain a homogeneous solution.
29. Weigh 20 mg of MnTCPP and add it to the solution, and sonicate for 5 min to obtain a homogeneous solution.
30. In a separate beaker, weigh 5mg of Ru NPs and add 5 mL of DMF to disperse the Ru NPs. Sonicate to obtain the Ru NPs dispersion.

***Note:*** The order of reagent addition should be consistent to avoid premature reactions. An ice pack can be added to the sonication bath to prevent DMF from overheating.

31. Combine the precursor solution and the Ru NPs dispersion, sonicate for 10 min to obtain a homogeneous Ru@PCN reaction mixture.
32. Transfer the Ru@PCN reaction mixture to a 50 mL PTFE liner, place it in a stainless-steel autoclave, and tighten the safety cap.
33. Place the autoclave in a 90°C oven and react for 5 h.

***Note:*** Wear heat-resistant gloves when handling the autoclave to prevent burns. Do not attempt to open the autoclave while under high temperature and pressure.

34. After the reaction, turn off the oven and open the door to dissipate heat for 1 h. Remove the autoclave to a safe area and allow it to cool naturally for ≥6 h before opening to obtain Ru@PCN product.

### Purification and freeze-drying of Ru@PCN NPs

**Timing: 6–7 days**

The following steps describe the purification and freeze-drying process for Ru@PCN NPs, which ensures the removal of unreacted reagents and solvents, resulting in pure Ru@PCN NPs powder suitable for further applications.

35. Transfer the Ru@PCN product solution to a 50 mL centrifuge tube and centrifuge at 15,292 × g (12,000 rpm), 4°C for 30 min. Discard the supernatant and retain the pellet.
36. Add sufficient DMF to the pellet and sonicate for 3 min to disperse. Centrifuge again (15,292 × g, 4°C, 30 min) and discard the supernatant to complete the first DMF wash.
37. Repeat steps 36 for a total of three DMF washes.

***Note:*** Once the centrifugation time is complete, immediately remove the 50 mL centrifuge tube and invert it to prevent the pellet from re-dissolving, which could result in product loss. If the tube is not inverted in time, perform a second centrifugation on the supernatant and retain the pellet.

38. Add deionized water to cover the pellet, sonicate for 5 min, and let stand for 2 h. Then centrifuge (15,292 × g, 4°C, 30 min) and discard the supernatant.
39. Add 10 mL deionized water to the pellet and sonicate for 5 min to obtain a uniform dispersion.
40. Transfer the dispersion to a 100 kDa dialysis bags and place it in a 1 L beaker containing 600 mL DI water with a magnetic stir bar. Dialyze at 600 rpm for 4 days.

***Note:*** Before use, carefully check the dialysis bag and dialysis clamp for airtightness to prevent leakage of the reaction solution, which could result in loss. The volume of solution added to the dialysis bag should not exceed half of its total volume to avoid low dialysis efficiency or rupture due to insufficient buffer space.

41. Replace the external DI water 3–4 times per day to maintain the concentration gradient and ensure efficient dialysis.
42. After dialysis, transfer the contents of the dialysis bags to a 50 mL centrifuge tube and centrifuge (15,292 × g, 4°C, 30 min). Discard the supernatant to obtain the purified pellet.
43. Pre-cool the purified pellet by placing the centrifuge tube at 4°C for 2 h, then transfer the tube to a −20°C freezer and pre-cool for 5 h. Afterward, transfer the tube to a −80°C freezer and freeze for 12–16 h.

***Note:*** Ensure sufficient pre-cooling time to prevent the formation of large crystals during freeze-drying, which could damage the nanoparticle structure.

44. Place the frozen sample in a lyophilizer and lyophilize for 2–3 days to obtain the final Ru@PCN NPs powder.

### Synthesis of Ru@PCN@PEI NPs

**Timing: 13 h**

This section describes the conjugation of polyethylenimine (PEI) onto Ru@PCN nanoparticles using the EDC/NHS coupling method.

45. Weigh 20 mg of Ru@PCN NPs and disperse in 10 mL of DI water by sonication for 5 min to obtain the Ru@PCN suspension.
46. In a separate 5 mL EP tubes, weigh 500.85 mg of polyethylenimine (PEI, Mw 1,800) and dissolve it in 2 mL DI water to obtain the PEI solution.
47. To the PEI solution, add 19.2 mg of 1-ethyl-3-(3-dimethylaminopropyl) carbodiimide hydrochloride (EDC·HCl) and 11.54 mg of N-hydroxysuccinimide (NHS) and mix until fully dissolved to obtain the EDC/NHS solution.
48. Place the Ru@PCN suspension on a magnetic stirrer (600 rpm) and, using a 1 mL pipette, slowly add the prepared 2 mL EDC/NHS solution to the suspension.

***Note:*** Maintain a stirring rate of approximately 600 rpm during the addition to prevent aggregation.

49. Stir the reaction mixture at 600 rpm and room temperature (25°C ± 1°C) for 12 h to obtain the surface-modified Ru@PCN@PEI suspension.

***Note:*** The stirring time can be adjusted as needed, but it must be at least 12 h.

### Purification and freeze-drying of Ru@PCN@PEI NPs

**Timing: 6–7 days**

The following steps outline the purification and freeze-drying process for Ru@PCN@PEI nanoparticles, ensuring the removal of unreacted reagents and solvents, and resulting in pure Ru@PCN@PEI nanoparticle powder suitable for further applications.

50. Transfer the Ru@PCN@PEI suspension to a 50 mL centrifuge tube and centrifuge at 15,292 × g (12,000 rpm), 4°C for 30 min. Discard the supernatant and retain the pellet.
51. Add 10 mL DI water to the pellet and sonicate for 3 min to re-disperse.
52. Repeat centrifugation and deionized-water washes (15,292 × g, 4°C, 30 min) 3 times to remove unreacted PEI, EDC, and NHS.
53. Disperse the purified pellet in 10 mL DI water to obtain a uniform Ru@PCN@PEI dispersion.
54. Purify and lyophilize the Ru@PCN@PEI NPs.
  a. Transfer the Ru@PCN@PEI dispersion toa 100 kDa dialysis bags and place it in a 1 L beaker containing 600 mL DI water with a magnetic stir bar. Dialyze at 600 rpm for 4 days.
  b. Replace the external DI water 3–4 times per day to maintain the concentration gradient and ensure efficient dialysis.
  c. Transfer the contents of the dialysis bags to a 50 mL centrifuge tube and centrifuge (15,292 × g, 4°C, 30 min). Discard the supernatant to obtain the purified pellet.
  d. Pre-cool the purified pellet by placing the centrifuge tube at 4°C for 2 h, then transfer the tube to a −20°C freezer and pre-cool for 5 h. Afterward, transfer the tube to a −80°C freezer and freeze for 12–16 h.
  e. Place the frozen sample in a lyophilizer and lyophilize for 2–3 days to obtain the final Ru@PCN@PEI powder.

### Characterization of Ru@PCN@PEI NPs and evaluation of SOD-like activity

**Timing: 2–3 h**

After the successful preparation of Ru@PCN@PEI NPs, scanning electron microscopy (SEM), transmission electron microscope (TEM) and dynamic light scattering (DLS) to characterize the morphology and particle size of Ru@PCN@PEI NPs. Additionally, the SOD-like activity of the Ru@PCN@PEI NPs is evaluated using the pyrogallol assay, measured by UV-Vis spectrophotometry.

55. Disperse 1 mg Ru@PCN@PEI NPs into 1 mL DI water and sonicate for 5 min to obtain a homogeneous dispersion.
56. Drop 20 μL of the Ru@PCN@PEI NPs dispersion onto a copper foil, then transfer the foil to an oven, and dry at 60°C for 1 h for TEM characterization.

***Note:*** TEM imaging should be performed by a trained technician or engineer following standard operating procedures.

57. Add 3 mL of DI water to the solution from step 55, mix by pipetting with a 1 mL pipette, then transfer the solution to a DLS cuvette for DLS measurement on the instrument.

***Note:*** Clean the DLS cuvette with ultrapure water, then rinse it three times with the sample solution. Afterward, fill the cuvette with the sample solution up to half its volume, sonicate to ensure uniform dispersion, and check if the solution is transparent and free of noticeable suspended or aggregated particles.

58. Prepare Ru@PCN@PEI NPs powder for SEM imaging, which will be performed by a professional engineer according to the standard SEM imaging procedure.
59. Preparation of 10 mM HCl
  a. Using a 100 μL pipette, transfer 83 μL of concentrated HCl (37%) into a 250 mL beaker.
  b. Add DI water to bring the total volume to 100 mL to obtain a 10 mM HCl.
  c. Aliquot the prepared 10 mM HCl into 50 mL centrifuge tubes, label them, seal with parafilm, and store at room temperature (25°C ± 1°C) in a cool, dry place.
60. Preparation of TE Buffer (50 mM Tris, 1 mM EDTA, pH 8.2)
  a. Weigh 606 mg of Tris and 29.2 mg of EDTA·2Na, add to a 250 mL beaker.
  b. Add 100 mL of DI water to the beaker and sonicate for 3 min until fully dissolved.
  c. Aliquot the prepared TE buffer into 50 mL centrifuge tubes, label them, seal with parafilm, and store at room temperature (25°C ± 1°C) in a cool, dry place.
61. Weigh 12.6 mg of pyrogallol and transfer it to a 15 mL centrifuge tube.
62. Using a 1 mL pipette, add 10 mL of 10 mM HCl and sonicate for 3 min to mix thoroughly, forming a 10 mM pyrogallol stock solution.

***Note*:** The pyrogallol is fully dissolved in 10 mM HCl to prevent self-oxidation, ensuring the activity of pyrogallol during subsequent SOD activity measurements.

63. Weigh 2 mg of Ru@PCN@PEI NPs powder and add it to 10 mL of TE buffer to prepare a 200 μg/mL Ru@PCN@PEI aq.

***Note:*** The step 63 describes the preparation of a 200 μg/mL Ru@PCN@PEI NPs aq. For other concentrations, simply dilute by the same factor.

64. Using a 1 mL pipette, transfer 784 μL of the 200 μg/mL Ru@PCN@PEI aq into a cuvette and place it into a UV-Vis spectrophotometer.
65. Add 16 μL of the 10 mM pyrogallol stock solution into the cuvette to achieve a final concentration of 0.2 mM of pyrogallol.
66. Immediately after adding the pyrogallol, start the time-scan mode on the UV-Vis spectrophotometer, measure the absorbance at 320 nm, recording every 30 seconds for a total of 10 min.

***Note:*** Pyrogallol undergoes self-oxidation in alkaline solutions to produce superoxide anion radicals (·O_2_^-^) and semiquinones. These semiquinones react to form red phenols, which increase absorbance at 320 nm. In the presence of SOD-like activity, the red phenols react to form a colorless product, leading to a decrease in absorbance at 320 nm. Therefore, lower absorbance at 320 nm indicates higher clearance of ·O_2_^-^, reflecting enhanced SOD-like activity of the nanocatalyst.^6^

## Expected outcomes

This protocol outlines the control of particle size of PCN-222(Mn) NPs by adjusting the amount of BA as a competitive ligand (Table 1). TEM images confirm the successful preparation of rod-shaped, uniformly distributed PCN-222(Mn) NPs (Figure 1A). The structural identity of PCN-222 is further confirmed by PXRD analysis (refer to Figure S2). Reducing the BA concentration led to a decrease in nanoparticle size overall (Figure 1B). However, when the ZrOCl_2_: BA ratio was reduced to 1:14, with BA below 324.8 mg, irregular product morphology was observed with flocculent edges and incomplete formation. At a ratio of 1:10, significant aggregation of the product was noted (Figure 1A). Therefore, 324.8 mg of BA was determined to be the minimum required concentration for successful PCN-222(Mn) NPs synthesis. The appropriate particle size of PCN-222(Mn) NPs is crucial for the subsequent preparation of Ru@PCN@PEI NPs.

**Figure 2:**
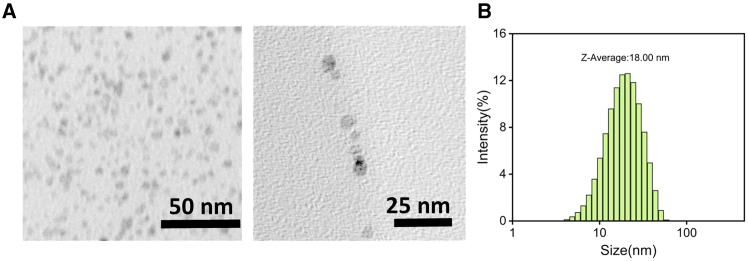
Characterization of Ru NPs (A) TEM imaging of Ru NPs. (B) DLS imaging of Ru NPs.

**Figure 3:**
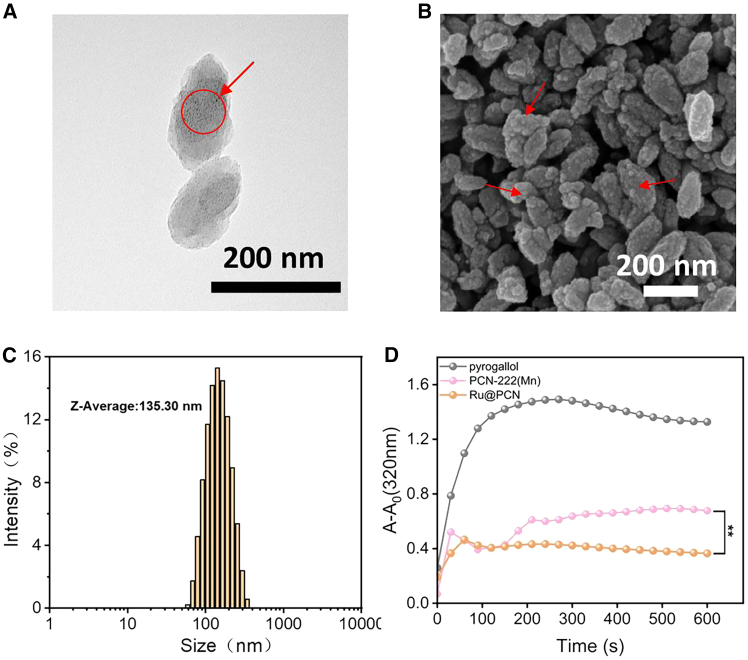
Characterization of Ru@PCN NPs (A) TEM imaging of Ru@PCN NPs (red arrows and circle indicate representative encapsulated Ru NPs). (B) SEM imaging of Ru@PCN NPs (red arrows indicate surface features corresponding to Ru loading). (C) DLS imaging of Ru@PCN NPs. (D) Comparison of SOD-like activity between Ru@PCN NPs and PCN-222(Mn) NPs (Data are presented as the mean ± SD (n = 3). Statistical significance was determined using one-way ANOVA (P < 0.01).∗∗).

**Figure 4:**
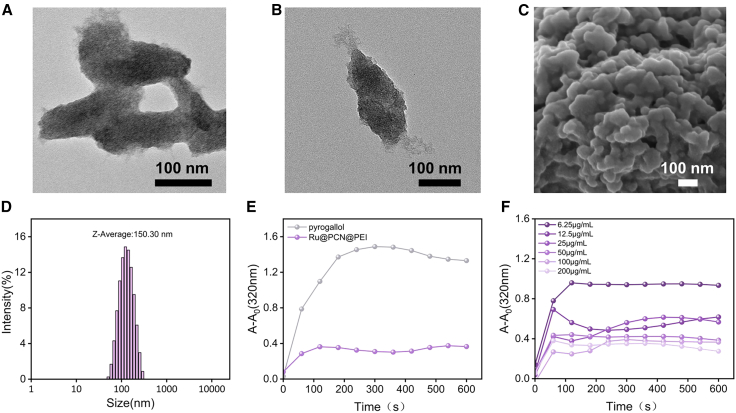
Characterization of Ru@PCN@PEI NPs and evaluation of SOD activity (A) TEM imaging of Ru@PCN@PEI NPs. (B) TEM imaging of Ru@PCN@PEI NPs. (C) SEM imaging of Ru@PCN@PEI NPs. (D) DLS imaging of Ru@PCN@PEI NPs. (E) UV-Vis time scanning spectrum of pyrogallol and Ru@PCN@PEI NPs at 320nm. (F) UV-Vis time-scanning spectra of Ru@PCN@PEI NPs at different concentrations at 320 nm.

After optimizing the particle size of PCN-222(Mn) NPs, Ru NPs were successfully synthesized via high-temperature reduction (Figure 2). These were subsequently loaded into the PCN-222(Mn) framework using a one-pot method to form Ru@PCN NPs. TEM and SEM images show that Ru NPs were uniformly loaded into the pores of PCN-222(Mn) NPs (Figures 3A and 3B), with minimal change in the size of the final Ru@PCN NPs (Figure 3C). The addition of Ru NPs significantly enhanced the SOD-like catalytic activity of PCN-222(Mn) NPs (Figure 3D). Following surface modification with PEI, the Ru@PCN@PEI NPs were obtained. The morphology and size characterization of Ru@PCN@PEI NPs are shown in Figures 4A–4D. The SOD-like activity of the nanoparticles was assessed using the pyrogallol assay, showing good catalytic activity and concentration dependence (Figure 4E and 4F). The uniform distribution of elements in Ru@PCN@PEI NPs can be further confirmed by EDS mapping (Figure S3).

## Limitations

While this protocol provides a reliable method for synthesizing Ru@PCN@PEI NPs and can be applied to the loading of other nanoparticles and polymer modification, several limitations should be considered. The synthesis of Ru@PCN NPs (without PEI modification) must be carried out in an autoclave, as this step is critical for determining the final morphology and particle size of the Ru@PCN@PEI NPs. Alternative reaction conditions, such as using an oil bath, may lead to different nanoparticle sizes and morphologies, making the reaction pressure an essential factor for consistent results. Additionally, this protocol is optimized for a 20 mL reaction volume, and its scalability to larger reaction volumes has not been explored. Larger reaction systems could introduce challenges in maintaining uniformity and controlling nanoparticle size.^7^

Regarding reaction yield, the protocol has not been systematically studied for yield efficiency, but based on the 20 mL reaction system, the yield after freeze-drying is approximately 20 mg, indicating a relatively low yield.^8^ The amount of benzoic acid (BA) used during synthesis affects the yield, and careful recovery of the product during centrifugation is necessary to minimize loss and avoid waste. Furthermore, when applying this method to modify other nanoparticles and polymers, it is essential to ensure that the nanoparticle size is small enough to enter the pores of the BMOF. Additionally, water-soluble polymers are preferred for surface modification, as they help maintain good dispersion in aqueous solutions.^9^

Additionally, while the current protocol ensures successful loading of Ru NPs, the precise incorporation efficiency has not been systematically quantified in this study. Detailed quantification and further optimization of the loading capacity remain to be explored.

## Troubleshooting

### Problem 1

The synthesized PCN-222 (Mn) nanoparticles do not have a rod-cone shape and the particle size is not ideal (steps 3–5 in “Synthesis of size-tunable PCN-222(Mn) NPs”).

### Potential solution

Ensure that the reactants, including ZrOCl_2_·8H_2_O, BA, and MnTCPP, are added in the exact order specified in the protocol. After each addition, thoroughly mix the solution before adding the next reactant to ensure uniform dispersion. Inadequate mixing or incorrect addition order may affect the nanoparticle synthesis process, leading to changes in morphology.

The reaction must be conducted under high-pressure conditions. This protocol uses an autoclave to maintain these conditions. Prior to starting the reaction, ensure that the autoclave is securely tightened to guarantee stable high-pressure conditions. If an alternative reaction vessel is used, make sure that the pressure conditions meet the necessary high-pressure requirements for the synthesis.^10^

Additionally, maintaining the optimized 90°C temperature within the high-pressure autoclave is critical to ensure the formation of the desired rod-shaped morphology and prevent the growth of other competing phases.

### Problem 2

Failure to load Ru NPs (steps 30–33 in “Synthesis of Ru@PCN NPs”).

### Potential solution

Check the particle size of the synthesized Ru NPs. The particle size should not be too large; if it is, prepare the Ru NPs again with a smaller size. This guideline applies to the loading of other nanoparticles as well.

Ensure that an adequate amount of Ru NPs is added in step 30, and that the mixture is sonicated thoroughly. Insufficient Ru NPs or uneven dispersion can lead to failure in nanoparticle loading. If necessary, increase the concentration of the Ru NPs solution to improve the loading efficiency.

### Problem 3

Difficulty in PEI surface modification (steps 46–47 in “Synthesis of Ru@PCN@PEI NPs”).

### Potential solution

Ensure the activity of the PEI stock solution. If possible, prepare it fresh just before use to ensure maximum effectiveness. The amounts of NHS and EDC can be adjusted as needed to ensure successful surface coating of PEI on the nanoparticles.

Make sure that the PEI solution is added slowly to the stirring Ru@PCN suspension. Rapid addition may result in poor dispersion, which can affect the coating efficiency and experimental results. If issues persist after addressing the above points, consider extending the reaction time to 24–48 h to improve the surface modification.

### Problem 4

The amount of precipitate (product) is low after centrifugation of the Ru@PCN@PEI NPs reaction solution (Step 50 in “Purification and Freeze-Drying of Ru@PCN@PEI NPs”).

### Potential solution

If the amount of precipitate is low after the first centrifugation, the primary failure mode is insufficient centrifugation. The grafting of PEI significantly alters the surface charge and dispersibility of the nanoparticles, which enhances their stability in the supernatant. Consequently, when centrifugation conditions (such as speed or equipment capacity) are limited, collect the supernatant and perform 2–3 additional rounds of centrifugation.^11^ After each centrifugation, combine the precipitate and proceed with the next steps. Alternatively, multiple reactions (2–3) can be run simultaneously, and the reaction solutions can be pooled together before centrifugation to increase the amount of precipitate.

### Problem 5

Aggregation of Ru@PCN@PEI NPs powder during re-dispersion after freeze-drying (Step 53 in “Purification and Freeze-Drying of Ru@PCN@PEI NPs”).

### Potential solution

The aggregation may be due to incomplete dispersion of the nanoparticles or insufficient incubation time with the aqueous solution. After washing the reaction product with deionized water, add 10 mL of deionized water to the Ru@PCN@PEI NPs pellet, sonicate for 5 min, and let it stand for 2–3 h to ensure uniform dispersion and adequate contact in deionized water. Additionally, the pre-cooling step should be performed gradually by keeping samples at 4 °C for 2 h and −20 °C for 5 h before transferring them to −80 °C for at least 12 h. This avoids abrupt cooling, which can damage the nanoparticle crystal structure and impair their dispersion properties.^12^

### Problem 6

Particle aggregation or uneven distribution observed during TEM characterization (steps 8–9 in “Characterization of PCN-222 (Mn) NPs”).

### Potential solution

The drop-casting method may lead to artificial aggregation due to the solvent evaporation process (coffee-ring effect). It is recommended to prepare a series of dispersions with different concentrations before sample deposition. The optimal dilution can be determined by comparing the resulting TEM images to ensure well-dispersed particles. Additionally, briefly sonicate the dispersion for 3–5 min immediately before casting to minimize any pre-existing soft aggregates.

## Resource availability

### Lead contact

Further information and requests for the resources are available from the lead contact, Xujie Liu (liuxujie@gdut.edu.cn).

### Technical contact

Further information and requests for the technical specifics of performing the protocol are available from the technical contact, Meijing Wang (wangmeijing1@mails.gdut.edu.cn).

### Materials availability

This study did not generate unique materials.

### Data and code availability

This study did not involve any code, and the data would be available upon request.

## Acknowledgments

This work was supported by the National Natural Science Foundation of China (32171314) and Guangdong Basic and Applied Basic Research Foundation (2024A1515011666).

## Author contributions

X.L. conceived and supervised the project. M.W. designed and performed the experiments and drafted the manuscript. T.C., X.Z., and J.L. assisted in the progress of the experiment. X.L. and Y.H. reviewed and edited the manuscript.

## Declaration of interests

The authors declare no competing interests.
