# Supplementary material for: Protocol for the synthesis and characterization of Ru@PCN@PEI nanoparticles with superoxide dismutase catalytic activity
Source: STAR Protoc. 2026 Mar 5;7(1):104412. doi: 10.1016/j.xpro.2026.104412 (PMC12992955; doi:10.1016/j.xpro.2026.104412)
Supplement: Document S1. Figures S1–S3 [file mmc1.pdf]

Supplemental Information

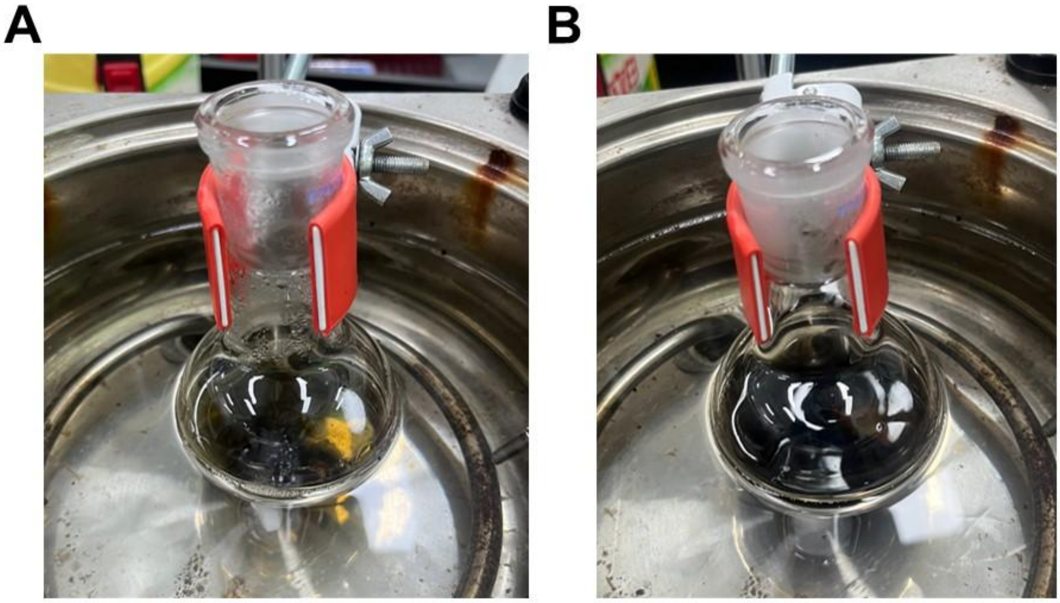

Figure S1. Color change during Ru NPs synthesis, Related to step 16. (A) Photograph of the reaction mixture at the initial stage, showing a characteristic yellowish-brown color. (B) Photograph of the deep-black Ru NPs suspension obtained after the addition of ascorbic acid and a 30 min reaction period.

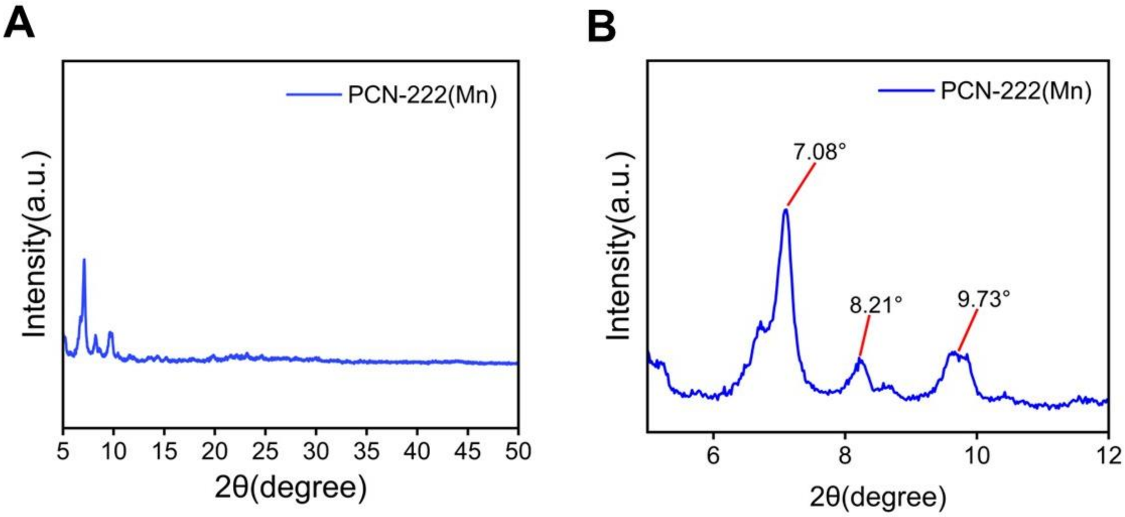

Figure S2. PXRD patterns and phase purity of PCN-222(Mn), Related to Figure 1 and Expected outcomes. (A) Wide-range PXRD pattern. The pattern was collected in the 2θ range of 5° to 50°, showing the high crystallinity and absence of amorphous or impurity phases in the bulk sample. (B) Zoomed-in PXRD pattern (5°–12°). The characteristic diffraction peaks observed at 2θ = 7.08°, 8.21°, and 9.73° are in high agreement with the reported hexagonal PCN-222(MOF-545) topology[S1], confirming the phase purity of the synthesized PCN-222(Mn) nanoparticles.

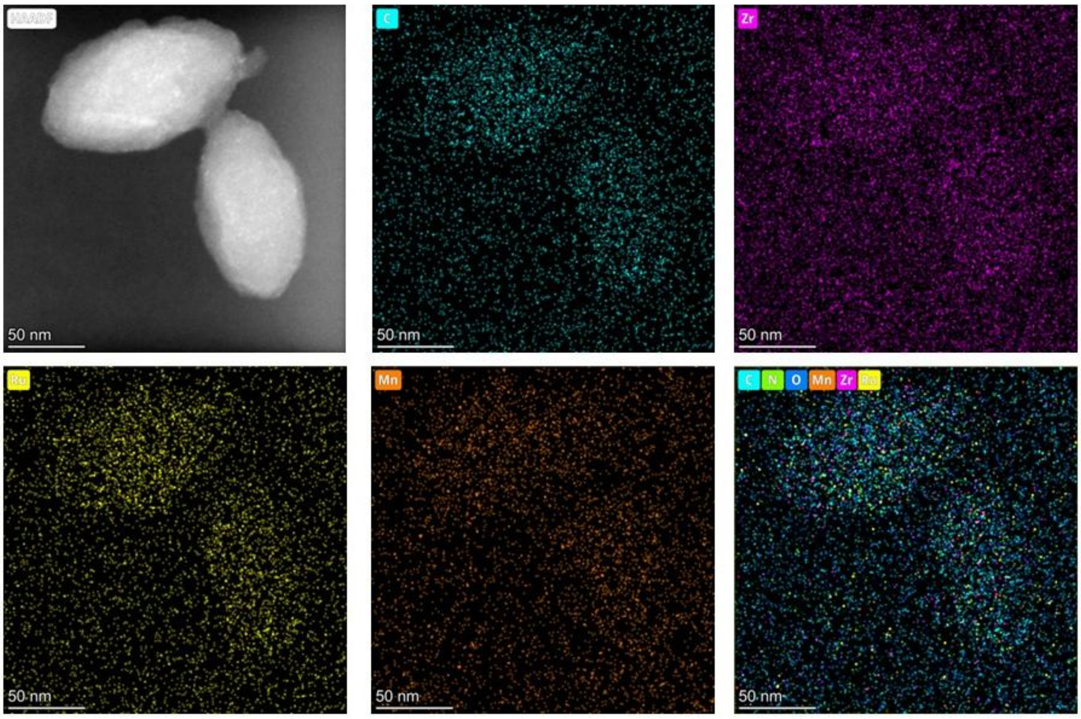

Figure S3. EDS elemental mapping of Ru@PCN@PEI NPs, Related to Expected outcomes. HAADF-STEM image and corresponding elemental distribution of C, Zr, Ru, and Mn, along with a merged elemental mapping image.

Supplemental references

[S1] Feng, D., Gu, Z.Y., Li, J.R., Jiang, H.L., Wei, Z., and Zhou, H.C. (2012). Zirconium-metalloporphyrin PCN-222: mesoporous metal-organic frameworks with ultrahigh stability as biomimetic catalysts. Angew. Chem. Int. Ed. Engl. 51, 10307-10310. 10.1002/anie.201204475
